# 398. Multicenter Evaluation of Outcomes of SARS-CoV-2 Positive Patients Treated at Rural vs Urban Hospitals in the United States

**DOI:** 10.1093/ofid/ofab466.599

**Published:** 2021-12-04

**Authors:** Karri A Bauer, Kalvin Yu, Vikas Gupta, Laura A Puzniak

**Affiliations:** 1 Merck & Co, Inc, Kenilworth, New Jersey; 2 Becton, Dickinson and Company, Franklin Lakes, New Jersey; 3 Merck & Co., Inc., Kenilworth, New Jersey

## Abstract

**Background:**

The SARS-CoV-2 pandemic has revealed socioeconomic and healthcare inequities in the US. With approximately 20% of the population living in rural areas, there are limitations to healthcare access due to economic constraints, geographical distances, and provider shortages. There is limited data evaluating outcomes associated with SARS-CoV-2 positive patients treated at rural vs. urban hospitals. The aim of the study was to evaluate characteristics and outcomes of SARS-CoV-2 positive patients treated at rural vs. urban hospitals in the US.

**Methods:**

This was a multicenter, retrospective cohort analysis of adult (≥ 18 years) hospitalized patients from 241 US acute care facilities with >1 day inpatient admission with a discharge or death between 3/6/20-5/15/21 (BD Insights Research Database [Becton, Dickinson & Company, Franklin Lakes, NJ]), which includes both small and large hospitals in rural and urban areas. SARS-CoV-2 infection was identified by a positive PCR or antigen during or < 7 days prior to hospital admission. Descriptive statistics were completed. *P* value of ≤0.05 was considered statistically significant.

**Results:**

Overall, 42 (17.4%) and 199 (82.6%) of hospitals were classified as rural and urban, respectively. A total of 304,073 patients were admitted to a rural hospital with 12,644 (4.2%) SARS-CoV-2 positive. In comparison, a total of 2,844,100 patients were treated at an urban hospital with 132,678 (4.7%) SARS-CoV-2 positive. Patients admitted to rural hospitals were older compared to those treated at an urban hospital (65.2 ± 17.3 vs. 61.5 ± 18.7, *P*=0.001) (Table 1). Patients treated at an urban facility had significantly higher rates of ICU admission, severe sepsis, and mechanical ventilation. ICU length of stay was significantly longer for patients admitted to an urban hospital compared to a rural hospital (8.1 ± 9.9 vs. 6.1 ±7.2 days, *P*=0.001) (Table 2). No difference in mortality was observed.

Table 1. Characteristics of SARS-CoV-2 positive patients treated at rural vs. urban hospitals.

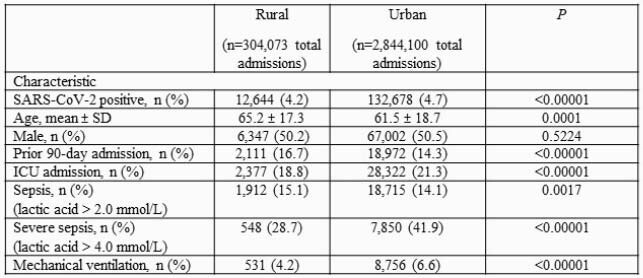

Table 2. Outcomes of SARS-CoV-2 patients treated at rural vs. urban hospitals. *Patients with available data.

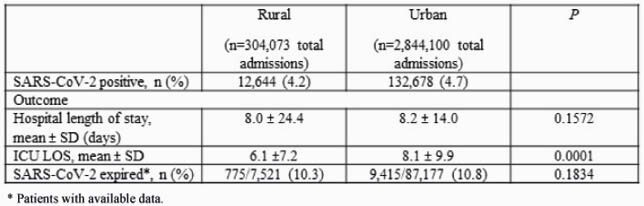

**Conclusion:**

In this large multicenter evaluation of hospitalized patients positive for SARS-CoV-2, there were significant differences in patient characteristics. There was no observed difference in mortality. These findings are important in evaluating the pandemic’s impact on patients in rural and urban healthcare settings.

**Disclosures:**

**Karri A. Bauer, PharmD**, **Merck & Co., Inc.** (Employee, Shareholder) **Kalvin Yu, MD**, **BD** (Employee) **Vikas Gupta, PharmD, BCPS**, **Becton, Dickinson and Company** (Employee, Shareholder) **Laura A. Puzniak, PhD**, **Merck & Co., Inc.** (Employee)

